# Cholelithiasis from the Surgical Point of View1Read before the Allegany County Medical Society, October 19, 1900.

**Published:** 1901-01

**Authors:** Eugene A. Smith

**Affiliations:** Buffalo, N. Y., Adjunct professor of clinical surgery, University of Buffalo


### CHOLELITHIASIS FROM THE SURGICAL POINT OF VIEW.[1]

By EUGENE A. SMITH, M. D., Buffalo, N. Y.,

Adjunct professor of clinical surgery, University of Buffalo.

IN THE past twenty years, remarkable advances have been made in abdominal surgery. Some men attribute this progress to technique alone, claiming that the brilliant results obtained by our leading abdominal surgeons have been attained by asepsis in operative work in conjunction with skill in operative procedures. Others ascribe it to accuracy of diagnosis, which is more and more sought for and with newer methods of clinical examination, and new lines of thought is more often attained. Granting to operative technique and accurate diagnosis their full measure of credit, it seems to me that we must give a fair proportion of credit for progress in abdominal surgery first to antiseptic measures summed up in the word drainage, which we employ in a large number of abdominal cases, and second, to the application of mechanical procedures in the operative treatment of diseased conditions interfering mechanically with the integrity of one or more organs or the functions of such organs.

Surgery has made its advance along these lines in the treatment of traumatism of the viscera, of appendicitis, of peritonitis, of obstructive and ulcerative conditions affecting the bowel, of ulcers and malignant disease of the stomach, and of inflammatory and obstructive disorders of the gall-bladder and its ducts.

It is no discredit to internal medicine to say that its field is limited when sepsis or traumatism, or disease producing mechanical inter-

---

1. Read before the Allegany County Medical Society, October 19, 1900.

ference with an organ or its function becomes threatening to life. While surgical treatment shows a high rate of mortality in grave cases coming under this classification, internal medicine offers practically no hope to the patient. The borderland cases between those coming properly within the province of medical treatment and those of such a nature as to belong unquestionably to the surgeon are on debatable ground still in many instances, and the physician is fortunate who makes no mistakes in adopting the proper line of treatment in all cases of abdominal disease.

In discussing cholelithiasis we find a subject broad enough to admit of discussion from the medical and surgical points of view. No one presumes to say that gallstones in the gall-bladder always call for surgical treatment, but no one can deny that numerous cases of suppurative cholecystitis and of gallstone cholemia have gone unrecognised and unoperated to a fatal termination, when surgery might have saved the patients. Judging from my own experience, the cases which clearly require operative interference to save life in a first attack of cholecystitis or biliary colic, with or without jaundice, must be rare. On the other hand, some practitioners overlook the fact that grave danger exists in these cases, and in a certain proportion of them this view results in tipping the balance against the patient, because the physician fails to urge timely surgical treatment in the cases that are beyond the reach of medical treatment.

Before we proceed to the discussion of the indications for operation in cholelithiasis, let us consider briefly the diagnosis of this condition from the surgeon's point of view. It is not necessary to rehearse the cardinal features of biliary colic, nor to dilate upon the significant appearances of icterus and cholemia when a plain history of frequent attacks of biliary colic can be obtained. Of cholecystitis it is sufficient to say it is easy to diagnosticate if jaundice is present after repeated attacks of biliary colic, and the patient is also acutely ill with fever, chills, digestive disturbance and a painful swelling in the right hypochondrium.

But when jaundice slowly develops without characteristic attacks of biliary colic, yet with painful attacks simulating biliary colic, diagnosis as to gallstones may be obscure. In one such case, seen with Dr. W. H. Mansperger, there was notable tenderness and rounded pyriform enlargement in the region of the gall-bladder. The patient was a man fifty-five years old, who complained of illness of six weeks' duration, beginning with jaundice, but who had had colicy pain on the right side at times for more than twenty years. He gained in weight for a time under Carlsbad treatment. After a few weeks, however, jaundice deepened and tenderness became more marked, stools were clay-colored, urine coffee-colored showing bile pigment, temperature was raised, leucocytosis not present. He fell off a few pounds in weight and became weaker. With a diagnosis of possible cancer, operation was undertaken to relieve the jaundice by a cholecystenterostomy. I found cancer of the omentum involving the gall-bladder. The operation gave no relief, the patient dying eight days later of cholemia.

In acute cholecystitis without jaundice the diagnostician is likely to be led astray. I have operated upon two cases which were diagnosticated to be appendicitis and were treated accordingly. The history of the more difficult of the two as regards diagnosis is as follows:

Mrs. T., aged 32, mother of two children, nursing a six months baby, had always had good health, except occasional attacks of indigestion with colicy pain, mainly referable to the right side, but with no history of jaundice. On September 4, 1900, her physician, Dr. E. E. Koehler, found her in one of her attacks, having acute pain most marked on the right side low down, with vomiting, constipation and excessive tenderness at McBurney's point, temperature and pulse raised. On his first visit he detected an obscurely outlined rounded mass, which muscle spasm and rigidity made vague, and which thereafter he could not feel, nor could I upon September 7th upon seeing her in consultation, till the patient was anesthetised. I agreed in the diagnosis of probable acute appendicitis with pus formation, temperature being 103°, pulse 120, respirations 32. Under chloroform ready for operation I found the mass pyriform, base entering the right inguinal region well below a line from the navel to the anterior superior spine of the ilium, the broad bulging body not felt at McBurney's point, and a neck extending upward to the region of the gall-bladder. The diagnosis was thereupon altered to that of sepsis, involving the gall-bladder or the appendix. Operation revealed a suppurative cholecystitis. The free border of the liver was found an inch below the free border of the ribs. The pyriform cholecyst was distended with glairy seropurulent fluid, more thickly purulent low in the sac and in the cystic duct, and not bile-stained. This sac-like mass projected well below the liver edge and was movable upward, inward and outward, somewhat resembling a floating kidney. Over seventy large and small calculi were taken from the gall-bladder, and bile began to flow upon removing a large stone lodged firmly in the cystic duct. The appendix having a distinct kink due to an adhesion, was also removed. In examining her scar on October 25, 1900, I found it to be three inches long, one-half extending above and one-half below a line drawn from the navel to the anterior superior spine of the ilium, and with its upper end two inches from the free border of the ribs. The incision indicates the spot where the mass was prominent and also the operator's uncertainty as to the condition to be attacked. In this case it was possible to make an appendectomy and cholelithotomy through one reasonably short incision, followed by drainage of a septic gall-bladder.

In dropsy of the gall-bladder due to occlusion of the cystic duct by calculi, a condition resembling floating kidney is found, and one patient found her way to me for operation for floating kidney sent by her physician, who had treated her for numerous attacks of colic, which he considered renal, but which were doubtless biliary. In this case moderate icterus was present and the tumor was pyriform, could not be moved downward on palpation, apex was up and was continuous with liver at the site of the gall-bladder under the free border of the ribs.

In a second case, which I saw with Dr. Carlton R. Jewett in June, 1900, he had excluded floating kidney, partly by the history of occasional jaundice and biliary colic, and cholelithiasis was further verified by sifting the feces and finding several small calculi. The tumor here was obscurely rounded, large, extending down to the line between navel and anterior superior spine and was tender. The patient was a tall, fleshy woman, 64 years old, and suffered constant right-sided pain, made more intense by lying on the left side. In operating I opened down on an enlarged right lobe of the liver, which proved to be the tumor, and under its margin I found an elongated gall-bladder full of inspissated bile and more than one hundred calculi, ranging from a pin-head to a bean in size. The largest ones were lodged in a sac-like dilatation of the cystic duct. My drain in this case upon closure of the wound emerged at McBurney's point.

The low situation of the gall-bladder in six of my cases, all of them women who had borne children, and the notable extreme low situation in the two cases just reported, leads me to believe that corsets and child-bearing must together influence this ptosis of the liver and gall-bladder, giving it a lower position than that assigned it in our anatomies.

When cholelithiasis with ulceration of duct or cholecyst has resulted disastrously in local or general peritonitis, diagnosis is at times much obscured. In a case of Dr. Mynter's, in which I was his assistant, duodenum, colon, stomach, gall-bladder and omentum were so matted together that cancer was diagnosticated after opening the abdomen. Death resulted some time later from sepsis, and a large calculus was found to have ulcerated through the common duct and to be the center of an inflammatory mass, in the center of which it lay surrounded by pus, the matted organs mentioned making up the supposed malignant tumor.

In discussing hepatic abscess, obstruction of the bowel, general peritonitis and malignant disease in the hepatic region, cholelithiasis must not be overlooked. In all these conditions it at times enters as an etiological factor likely to confuse and obscure diagnosis if not considered.

Turning now to a consideration of the indications for operation for cholelithiasis, we enter upon debatable ground. Is biliary colic a reason for cholecystotomy? The vast majority of patients suffering from a primary attack are not operated upon, and the conclusion is often drawn that operation is, therefore, not indicated. But death has been known to result from the shock produced by the passage of a biliary calculus. It may also be said that a certain number of cases after the first attack of biliary colic will develop immediately obstructive conditions in the ducts or septic conditions from infection, or even perforations of the ducts and consequent general peritonitis. In these cases the conservative practitioner must yield to the operator. Operation is also indicated if biliary colic recurs, the attack becoming more frequent and more severe. Outside of the question of dangerous sequelæ of gallstones in the gall-bladder, the attacks of colic are debilitating from the agony of body and distress of mind they entail. From another standpoint, Greig Smith asserts that in more than 80 per cent. of cases of cancer of the liver and gall-bladder and their ducts, calculi are found. While the relationship of cholelithiasis to cancer is not fully investigated, there seems reason to urge cholelithotomy in cholelithiasis to lessen the danger of the development of cancer.

Hydrocholecyst, or dropsy of the gall-bladder, due to obstruction of the cystic duct by stricture or calculus, may reach enormous size with dangerous thinning of the cyst walls and finally make operation highly necessary. The following case, seen with Dr. W. H. Mansperger, is extremely interesting to me, showing the obstructive effects, first of calculus, which was relieved by cholecystotomy, and second, of cicatricial development, which is at present causing trouble:

The patient, Mrs. D., aged 32, mother of six children, had had many severe attacks of biliary colic without jaundice, until April, 1900, when she had an attack followed by jaundice, just after her last confinement, the baby being three weeks old. On May 9, 1900, I made a cholecystotomy for moderate jaundice and pyriform tumor in the right hypochondrium. I found one gallstone the size of a large hickory nut occluding the cystic duct, as was proved by the glairy mucoid material contained in the gall-bladder. This fluid was like white of egg, not at all bile-stained, and distended the gall-bladder, making a sac the size of a large pear. Bile was discharged after the operation, but soon ceased, the jaundice passed away and the wound closed. A few weeks later the pyriform tumor reappeared and the same mucoid material was evacuated upon opening a pointing vesicle-like pouch in the line of the scar. The same fluid now constantly forms and at intervals of a few days needs opening to relieve pain. The patient opens the gall-bladder herself with a knitting needle when distention becomes painful. A probe can be swept around within the gall-bladder and meets with obstruction not due to calculus about five inches from the surface. In this case, cicatricial contraction at the site of the calculus which was removed has occluded the cystic duct. The patient is now considering the question of cholecystectomy, which I advise for the relief of her hydrocholecyst.

A frequent complication of cholelithiasis, arising from infection, is suppurative cholecystitis, leading to empyema of the gall-bladder. Among my cases of cholecystotomy, six in number, three were urgently necessary because of this condition, one of which I have already mentioned. The dangers here are septicemia, perforation or gangrene of the gall-bladder, massing and matting of adhesions with bowel obstruction, biliary fistulæ, general peritonitis and death. In this class of cases membranous shedding of the mucosa of the gall-bladder may occur, a condition which I found in the case of Mrs. S., operated upon September 9, 1897, at the German Deaconess's Home. The specimen of exfoliated mucosa, with perhaps some of the muscularis, I still have preserved in 1 per cent. formalin solution. In this case the calculi numbered six, four of them very large, each being the size of a hickory nut.

Biliary fistula, opening externally, occasionally follows suppurative cholecystitis, where fortunately for the patient pus points and is evacuated on the surface. This termination of bile tract infection by a natural cholelithotomy is rare, and should in no sense be urged as an argument in favor of nonoperative treatment. In the case just mentioned of suppurative cholecystitis with gangrenous exfoliation of the mucosa, a large calculus in the cystic duct was purposely left because of depth and immobility of the stone and the friable septic condition of the cholecyst. Later the stone could be readily and plainly touched with a probe deep in the gall-bladder. The biliary fistula refused to close and six months later I reoperated and removed the calculus after crushing it, finding, upon reassembling the fragments, a stone the size of a hickory nut. In this case the calculus had unquestionably worked toward the fundus of the gallbladder and nearer the surface and thus cholelithotrity was made much easier, at a time when reparative work had eliminated the dangerous features of sepsis present at the first operation.

Obstruction of the common duct by lodgement of a calculus also demands operation to overcome persistent icterus and to prevent the development of cholemia. In the case of Mrs. O., referred to me by Dr. J. S. Smith and operated upon April 25, 1900, at the Sisters' of Charity Hospital, lodgement of a calculus took place in the common duct, following the last of several severe attacks of biliary colic, characterised by icterus of brief duration. The last attack occurred in August, 1899, and the icterus lasting more than six months led to the development of eczema, by which the patient was driven to operation because of intolerable itching of arms, legs and body. Thirty stones were removed by cholecystotomy, one large one from the common duct. The fistula closed in two weeks, jaundice disappeared in three weeks and no itching has been noticed since. She lost most of her hair while jaundiced, and since the operation has recovered it. This same case illustrates the so-called "ball valve" action of a calculus, a condition which also calls for operative relief. In cases of this class, a calculus, which is too large to pass through the duct, becomes movable in a sac-like expansion of the duct due to the distention caused by backing of bile. Characteristic attacks of gallstone colic with accompanying jaundice recur.

Lastly, obstructive lodgement of calculi in the common duct may promote infection and suppurative cholangitis, the sequelæ of which are sepsis and hepatic abscess, conditions amenable only to surgical treatment.

The pathology of cholelithiasis is too extensive a subject to come within the scope of this paper, but it may be said that bacterial infection of the bile tracts is now considered to be the prime factor in etiology.

It is known that the colon bacillus is pus-forming, and under favoring circumstances, especially when mechanical interference with the biliary flow arises, it may lead to suppurative cholecystitis and empyema of the gall-bladder. Other infectious organisms, according to recent investigations, are found to lead to pus formation in the biliary tracts. When local conditions or the character of the bacteria lead to an infection of the catarrhal inflammatory type in the bile tracts, suppuration does not take place, but the deposit of cholesterin and bile salts about a focus of bacterial activity leads to gallstone formation. The colon bacillus and the typhoid bacillus have lately been found by numerous observers as the basis of biliary calculi.

When we consider cholelithiasis from this pathological point of view, the treatment is put upon a broad surgical plane that leaves to internal medicine the close study of the subject from the diagnostic standpoint with a view to the course to be followed in treatment. Treatment should be pursued along medical lines after diagnosticating cholelithiasis to the point of determining that its manifestations are threatening to the patient from the element of sepsis, or of mechanical interference with the biliary flow or the conjunction of these two elements. Internal medicine should then call upon surgery to drain for sepsis and to afford mechanical relief to mechanical interference with the biliary current.

In conclusion, at the risk of being tautological, I will say that to me it seems that the indications for operative treatment of cholelithiasis can be summed up under two headings: In the first place, operate for biliary calculus obstruction along the bile tracts, which has in its train of sequelæ recurring colic, hydrocholecyst, persistent jaundice and cholemia; in the second place, operate for sepsis of the bile tracts when it develops such dangerous conditions as suppurative cholangitis, suppurative cholecystitis, or perforation of the duct or cholecyst. In both classes of cases suffering and death are to be dreaded in the majority of cases more from the disease and its sequelæ than from the surgeon's knife.

1018 MAIN STREET.

---

